# HLA association with antipyretic analgesics-induced Stevens-Johnson Syndrome / toxic epidermal necrolysis with severe ocular surface complications in japanese patients

**DOI:** 10.1186/2045-7022-4-S3-P89

**Published:** 2014-07-18

**Authors:** Ryosuke Nakamura, Nahoko Kaniwa, Mayumi Ueta, Chie Sotozono, Emiko Sugiyama, Keiko Maekawa, Akiko Yagami, Setsuko Matsukura, Zenro Ikezawa, Kayoko Matsunaga, Katsushi Tokunaga, Michiko Aihara, Shigeru Kinoshita, Yoshiro Saito

**Affiliations:** 1 National Institute of Health Sciences, Japan; 2 National Institute of Health Sciences, Medicinal Safety Science, Japan; 3 Kyoto Prefectural University of Medicine, Department of Ophthamology, Japan; 4 Fujita Health University, School of Medicine, Department of Dermatology, Japan; 5 Yokohama City University, Graduate School of Medicine, Department of Environmental Immuno-Dermatology, Japan; 6 International University of Health and Welfare, Atami Hospital, Japan; 7 University of Tokyo, Graduate School of Medicine, Department of Human Genetics, Japan

## Background

Stevens-Johnson syndrome (SJS) and toxic epidermal necrolysis (TEN), are severe adverse drug reactions which are very rare, acute, serious, and potentially fatal.They exhibit characteristic symptoms not only in the skin, but also in the mucosal tissues such as ocular surface, oral cavity, and genitals. Although antipyretic analgesics (AAs) are some of the most frequent causative drugs of SJS/TEN, there has been no HLA association studies on these drugs-induced SJS/TEN. The scope of this study is to investigate HLA-type association with AA-related SJS/TEN with severe ocular surface complications (SOCs).

## Method

Japanese SJS/TEN patients were recruited to this study through the Japan Severe Adverse Reactions (JSAR) research group. The SJS/TEN patients can be divided into three groups: (i) patients with SOCs who received AA for relieving a common cold before the onset of SJS/TEN (20 cases); (ii) patients without SOCs who received AA for relieving a common cold before the onset of SJS/TEN (16 cases); (iii) patients with SOCs who received causative drug(s) for relieving other than a common cold (38 cases). HLA class I loci (HLA-A, C, and B) were genotyped for these patients and for healthy Japanese volunteers (n = 220). For these HLA genotypes, frequencies in the case groups were compared with those in the healthy controls.

## Results

Significant association of SJS/TEN with HLA-A*02:06 was found in the group (i), as previously reported (Ueta et al, Mol Vision 14:550 2008). In addition, we found significant association with HLA-A*33:03-C*14:03-B*44:03 haplotype in this group, which is the second most frequent haplotype in Japanese population (5.605%). Fourteen patients of the group (i) had been administered acetaminophen, and these two HLA-type/haplotype associations were still significant in the acetaminophen-administered subgroup of the group (i). On the other hand, these two associations were not significant either in the groups (ii) or (iii), although there was a small difference in the frequency of HLA-C*14:03-B*44:03 haplotype between groups (i) and (ii).

## Conclusion

In the Japanese population, we found that HLA-A*02:06 genotype and HLA-A*33:03-C*14:03-B*44:03 haplotype are independently associated with AA-induced SJS/TEN with SOCs. These biomarkers would be useful to discover mechanisms of and to develop a new therapeutic method for SOCs caused by AA-induced SJS/TEN.

